# Protocols and challenges in managing multi-source biological evidence at the crime scene: a scoping review

**DOI:** 10.1016/j.fsisyn.2026.100722

**Published:** 2026-08-10

**Authors:** Mayur Sudhir Balbudhe, B Suresh Kumar Shetty, Saroj Babar, Akshita Sinha, Sahil Gupta

**Affiliations:** a Department of Forensic Medicine and Toxicology, Kasturba Medical College Mangalore, Manipal Academy of Higher Education, Manipal, India; b School of Forensic Science, National Forensic Sciences University, Dharwad Campus, India; c Department of Forensic Science, Adamas University, Kolkata, India; d Study Mission High School, Kolkata, India

**Keywords:** Forensic DNA, Touch DNA, Trace DNA, Crime scene processing, Evidence preservation, Chain of custody, Trace evidence, Biological fluids, DNA degradation, Contamination control, Forensic protocols

## Abstract

To maximise DNA yield from evidence and ensure the integrity of the chain of custody, all biological evidence must be properly managed and preserved during initial crime scene processing. This scoping review aimed to collate documented protocols and issues associated with processing blood, semen, saliva, and touch DNA at the crime scene to optimise downstream DNA processing. A synthesis review of 57 peer-reviewed studies (2015-2025) was conducted using a curated table of forensic DNA evidence. Data were presented in a table organised by study features, protocols, problems, solutions, results, and suggestions. Findings from the studies referenced favour optimised double-swab (wet + dry) or adhesive tape-lifting techniques with buffered solutions (e.g., PBS, SDS) and visualisation aids (e.g., Diamond Nucleic Acid Dye); immediate air-drying or active-drying systems; chemical preservatives (Longmire's buffer, ethanol, solid NaCl); cold storage; and carrier additives. Strict anti-contamination procedures (PPE, elimination databases, negative controls) are essential, as are separate tamper-evident packaging. These protocols improve DNA recovery, reduce degradation and stochastic effects, and enhance success in STR and mtDNA profiling. Key challenges include low-template stochasticity, shedder variability, substrate interference, environmental degradation (especially aquatic and thermal), operator variability, and secondary transfer. Most studies have been conducted in laboratories, which may not be generalisable to real-world settings. Rapid, substrate-tailored collection with minimal handling, immediate preservation, and a strict chain of custody constitute the current best practice for evidence collection at the crime scene. Future studies should focus on multi-site field validations, automation, and harmonised guidelines to address current translational gaps and enhance equity in forensic contexts.

## 1 Introduction

Blood, semen, saliva, DNA trace (touch), DNA, and other biological evidence are among the most important items recovered from a crime scene, connecting an individual to an object, victim, or location and aiding downstream DNA profiling and the interpretation of activity [1]. However, the value of the recovered item for forensics largely depends on how it is processed at the initial scene, where contamination, moisture, heat, packaging contact, and unnecessary disturbance can quickly diminish its potential for recovery and compromise the integrity of the evidence [2].

The literature repeatedly suggests that before any effort is made at collection, a careful record, visual examination, photograph, and description of the biological evidence should be made first [[1], [2], [3]]. This is significant because the context of the stain (its spatial arrangement, size, and appearance) can be of interpretative value, as can the biological material [3]. Trace DNA evidence may be present in forms other than visible stains, including latent touch deposits, fibres, and other trace residues; documentation should therefore extend beyond the appearance of the evidence, also to record its condition, precise location, and the specific site selected for sampling. Once found, evidence should be gathered in a manner appropriate to the substrate and type of evidence, and secondary transfer and disturbance to the exhibit's original condition should be minimised [1].

Although relatively visible, blood evidence remains susceptible to degradation if the item is wet, exposed to changing environmental conditions, or improperly transported [4]. Blood and other biological fluids, as well as semen and saliva stains, can penetrate porous materials, such as fabrics, and persist for longer periods [5]. However, the amount and quality of recovered DNA may be compromised by degradation over time, dye interference, and substrate absorption [[6], [7], [8], [9]]. Because the amount of template is often very small, the chance of obtaining a useable profile is significantly compromised by small losses during swabbing, packaging, and transport to the laboratory [7,8]. Trace DNA is the most fragile category [6].

One of the most prevalent ideas in the literature is that there is no one-size-fits-all approach to collecting all biological evidence. Rather, the collection process should be based on the nature of the substrate and the condition of the sample, and should be selected from a continuum of procedures that include the use of a range of suitable swabs, drying techniques, packaging materials, and handling procedures, informed by risk assessment and the available evidence [6,9]. The chain of custody is critical and depends on proper labelling and sealing, separate packaging, and accurate documentation of all transfers from the scene to the laboratory. These steps can be combined to maximise downstream forensic yield while maintaining the legal admissibility of the evidence.

## 2 Methodology

A scoping review was conducted using the Arksey and O'Malley framework [10]. It was also complemented by the Joanna Briggs Institute (JBI) methodology (Aromataris et al., 2024 [11]) and the PRISMA-ScR checklist, which helped identify studies, concepts, and gaps. The methodology consisted of five steps: (1) defining the research question, (2) searching the literature, (3) selecting the literature, (4) charting the findings of the studies, and (5) synthesising, summarising and reporting the findings. The JBI updates are based on the latest PRISMA-ScR Guidance.

### 2.1 Research question

What protocols and challenges are documented for managing and preserving multi-source biological evidence (blood, semen, saliva, touch DNA) during the initial crime scene processing to maximise downstream forensic analysis yield and chain-of-custody integrity?

### 2.2 Identification of relevant studies

The literature search was performed in the Scopus, Embase and PubMed databases. A search strategy incorporating keywords and index terms was used (see Table 1). As ‘touch DNA’ and ‘trace DNA’ are used interchangeably in the forensic literature, the search strategy incorporated both terms; a supplementary search using ‘trace DNA’ was subsequently conducted and cross-checked against the original search results to confirm that no eligible studies had been missed. Only articles (published between 2015 and 2025) that were in English (or abstract with English) were included. No geographical restrictions were applied. Only data, opinion pieces, theses, and grey literature were excluded (see Table 2).

**Table 1:** Summary of the keywords used.

Broad Category	Keywords & Phrases
Biological Evidence	biological fluids crime scene, blood, semen, saliva, touch DNA
Collection & Preservation	crime scene processing, evidence collection, evidence preservation, collection protocols, preservation protocols
Challenges & Chain of Custody	challenges, contamination, chain of custody
Forensic Analysis	forensic analysis, DNA yield

**Table 2:** The findings from all relevant papers are summarised in the table below.

Sr.	Authors (Year, Region)	Evidence Type	Key Protocols	Main Challenges	Yield & CoC Strategies	Main Findings	Limitations
1	Jeromelj et al. (2025, Slovenia) [12]	Ancient petrous bone DNA	Chemical cleaning, diamond saw under LN_2_, powder grinding, qPCR (PowerQuant)	Unregulated temp/humidity fluctuations (5–35°C) over 12 years	Immediate post-excavation delivery, controlled 16–20°C/45–65% RH, strict anti-contamination	Significant reduction in DNA yield (p < 0.001) and increased degradation after long storage	Small fresh sample size, site-specific
2	Hymus et al. (2025, Australia) [13]	Trace/simulated DNA (fluorescein)	AutoLys tubes, adhesive films vs strip caps, centrifugation	Leakage from seals, condensation, and well-to-well transfer	Investigator chemistry, full 1-min spin, single-use lids	Adhesive films + proper centrifugation significantly reduce contamination risk	Fluorescein may not fully mimic DNA behaviour
3	TIPLAMAZ et al. (2025, Türkiye) [14]	Airborne human eDNA	Sterile cotton filters, oil-free vacuum, −20°C storage, Qiagen extraction	Very low nuclear DNA yield, mixed profiles, high contamination	Cotton filters preferred, mtDNA sequencing, PPE + negative controls	Successful mtDNA haplotypes; limited nuclear STR profiles	Small sample size, mixture interpretation issues
4	Andruszkiewicz Allan et al. (2025, USA) [15]	Aquatic eDNA (bottlenose dolphin)	3L volume, 5 μm filters, Longmire's/PCI extraction	High total DNA diluting rare target, inhibition	Larger volume + pore size, compatible preservative-extraction pairs	Improved target-to-total DNA ratio and detection	Single species/region
5	Saamia et al. (2024, Indonesia) [16]	Touch DNA on porous/non-porous ropes	Double-swab (wet + dry) or tape-lift, PrepFiler BTA	Shedder level variation, mixed profiles	Standardised hand-washing, controlled 22°C/70% RH	Shedder status significantly impacts yield and profile quality (p < 0.001)	Small n = 9, limited substrates
6	Zapico et al. (2024, USA/Germany) [17]	Saliva stains on fabrics	Cotton swabbing with SERATEC buffer, modified Qiagen DNeasy	Degradation over 90–180 days, dyed fabric interference	Additional extraction buffer, silica-based isolation	Full mtDNA profiles up to 180 days despite partial nuclear profiles	Indoor simulation, two donors only
7	Arsenault et al. (2025, UK) [18]	Trace/cellular & cell-free DNA on non-metals	Single wet-dry cotton swab with Elution Buffer	Porosity absorption, fungal contamination in humid conditions	Surrogate DNA, standardised swabbing, PPE in sterile rooms	DNA persisted up to 1 year; cfDNA better on non-porous surfaces	Swabbing may miss embedded DNA
8	Madden et al. (2024, Australia) [7]	Touch DNA on tape lifts	Diamond Nucleic Acid Dye staining, automated DNA-IQ extraction	Background fluorescence on fabrics	Microscopic cell counting (>500 cells threshold)	Cell count accurately predicts uploadable STR profiles	Specific to the lab workflow
9	Macías et al. (2024, USA) [19]	Burned human bone (femur)	Colour analysis (L*a*b*), light sanding, automated extraction	Severe degradation in calcined bones, subjective colour grading	Prioritise light/dark brown bones	Colour values predictive of DNA quantity and STR success	Small sample, automated kit
10	Oechsle et al. (2024, USA) [20]	Human DNA on cotton swabs	Salmon sperm DNA (spDNA) carrier, PrepFiler Express	High absorptive loss in swabs	10 μg spDNA carrier + automated extraction	Recovery improved from ∼29% to ∼100% without inhibition	Pristine pre-extracted DNA
11	Shahzad et al. (2024, Sweden/Pakistan) [4]	Aquatic (blood, touch, hair)	Police diver retrieval, comparative storage (dry, freeze, nitrogen)	Prolonged water submersion causes fragmentation	Immediate air-drying or −30°C freezing	mtDNA is highly robust; nuclear STR success drops sharply after 21 days	Static lake environment
12	Meakin et al. (2024, Australia) [21]	Trace/touch DNA	Applicator-assisted tape lifting	User contamination from handling	Applicator reduces direct contact	Applicator equal to or superior to the manual method	Lab-seeded samples
13	Griffin et al. (2023, Australia) [22]	Trace DNA on illicit drug powders	QIAamp DNA Investigator, centrifugation	Powder interference & co-elution	Optimal ∼200 mg powder mass	Investigator kit is superior for yield and purification	Saliva-spiked samples
14	Haarkötter et al. (2023, Spain) [23]	Degraded skeletal remains	Petrous bone prioritisation, organic phenol-chloroform	Long-term burial (70–80 yrs) degradation	1g powder, full demineralisation	The petrous bone yielded 15–30× more DNA than long bones	Organic extraction specific
15	Comment et al. (2023, Switzerland) [24]	Touch DNA (casework)	ISO 18385 swabs, single swabbing technique	Yield differences & handling practicality	Staff training, proper moistening	New swabs significantly outperformed the reference cotton	Local CSI practices
16	Elwick et al. (2022, USA) [25]	DNA on cartridge casings	Rinse-and-swab or soaking methods	Firing heat, copper inhibition	Chelators (EDTA/BTmix), rapid processing	Nickel-plated ≫ brass; unfired > fired	High analyst variability
17	Schulte et al. (2023, Switzerland) [6]	Touch DNA on glass	SDS or PBS moistened swabs, active drying	Low/variable shedding, storage degradation	Detergent solutions (∼45 μL)	SDS significantly higher yield; stable up to 12 months	Single good shedder
18	Harnpicharnchai et al. (2023, Thailand) [26]	Airborne eDNA	Custom high-volume multi-filter sampler	Low biomass, temporal variability	Cascade filter holders	Superior DNA yield and PCR quality vs commercial samplers	Specific low-biomass sites
19	Yuliarti et al. (2023, Indonesia) [27]	Neonatal buccal swabs	OraCollect kit, 4°C storage up to 14 days	Prolonged shipping in the archipelago	Cold chain, aliquoting	No significant difference in yield/purity after 14 days	Small n = 20 neonates
20	Ramírez de Arellano Sánchez et al. (2022, México/Germany) [28]	Bone samples (field)	Solid NaCl, EtOH-EDTA comparison	Warm/humid field conditions	Solid NaCl + complete cleaning	Solid NaCl gave the highest DNA yield and full STR profiles	Primarily a pig model
21	Holzschuh & Köpfli (2022, USA) [29]	Dried blood spots (DBS)	Whole blood vs DBS, magnetic beads	5–10× lower recovery in DBS	Whole blood preferred or optimised Tween-Chelex	Whole blood is superior for low-density detection	Lab mock samples
22	Stella et al. (2022, Australia/UK) [8]	DNA transfer in packaging	Double-swabbing of bags	Secondary transfer to packaging	Separate packaging, minimal movement	Frequent transfer affecting attribution	Small-scale experiments
23	Zareef et al. (2025, Pakistan) [30]	Subungual DNA (drowned)	Flocked swabs, phenol-chloroform	Rapid degradation in polluted water	Immediate single wet swab	Highest yield in tap water; recoverable to 48h	Synthetic prosthetic fingers
24	Le et al. (2025, Vietnam) [31]	Facial skin microbial DNA	Gentle blade scraping	Low biomass with swabbing	Scraping of the stratum corneum	Scraping yielded measurable bacterial/fungal DNA	Small pilot n = 10
25	Asiri et al. (2025, Saudi Arabia) [9]	Touch DNA (glass/phone)	Adhesive tape vs swabbing, direct PCR	Allele dropout in low-template	Adhesive tape + direct PCR	Tape achieved 85% complete profiles	Controlled lab conditions
26	Tomke et al. (2025, USA) [32]	eDNA (filters)	Longmire's/frozen preservation, Qiagen extraction	Inhibition with PCI, long-term degradation	Qiagen kit + cold storage	Highest yield and stable detection 1–4 years	Lab-based aquarium
27	Thong et al. (2025, Singapore) [33]	Semen stains	Saral Transfer Paper for AP-positive areas	Time-consuming documentation	Transfer paper for mapping	Faster documentation with minimal PCR impact	Cotton substrate only
28	Lisman et al. (2025, Poland) [34]	FFPE tissue	Maxwell RSC Xcelerate automated kit	Formalin-induced fragmentation	Automated extraction, DI assessment	Good yield but often partial STR profiles	Archival cancer samples
29	Lee & Syn (2025, Singapore) [35]	DNA transfer in evidence packages	Double-swabbing	Secondary transfer within packages	Pack items separately	Transfer in 39% of items, altered attribution in 10%	No movement/friction
30	Gaskell et al. (2025, UK) [36]	Environmental DNA in SARCs	Wet-dry double swabs, EM monitoring	High background DNA	PPE, elimination database, risk-based cleaning	Proper measures prevented contamination of evidential samples	Mock volunteer exams
31	Santamaria et al. (2025, UK) [37]	Sub-gingival plaque	Direct tube collection + beads	Pellet disturbance/loss	No-transfer method with ceramic beads	Significantly higher DNA yield and diversity	Periodontitis patients only
32	Kravanja et al. (2025, Slovenia) [38]	Petrous bone aDNA	Full demineralisation, EZ1 kit	Soil pH, temperature, and moisture	Prioritise dense petrous bone	Alkaline/cooler sites showed higher yield	Limited meteorological data
33	Priya & Sudhakaran (2025, India) [39]	Shrimp virus DNA	Novel DMSO-based extraction	Chitin-rich tissue, inhibitors	DMSO lysis + acetone precipitation	Highest yield, purity and sensitivity	Specific to muscle tissue
34	Zupanič Pajnič & Leskovar (2025, Slovenia) [40]	WWII bone powder	RT vs −20°C storage, yearly testing	Degradation in old bones	Homogenised powder	No significant difference up to 3 years at RT	Limited to poorly preserved bones
35	Castella et al. (2025, Switzerland) [41]	Indirect DNA transfer (case)	Medical swabs, differential extraction	Secondary transfer via shared items	Strict PPE and elimination databases	Blanket-mediated contamination misled the case	Single real case
36	Brandão Gontijo et al. (2025, Brazil) [42]	Tropical peat DNA	Optimised PowerSoil with phenol-chloroform	Humic acids, low pH	Increased sample weight + inhibitor removal	∼4× higher yield and better qPCR performance	qPCR validation focused
37	Samsonova et al. (2024, Russia) [43]	Dried blood on membranes	Glass fibre + spin-column elution	Membrane retention loss	Elution + lysis with membrane	50–60% recovery from glass fibre	Goat blood only
38	Brown et al. (2025, Multi-country) [44]	Insect voucher DNA	Non-destructive Chelex resin	Low yield from small specimens	Pre-lysis bleaching, no PK inactivation	Higher yield than silica columns	Single leafhopper species
39	Leskovar & Zupanič Pajnič (2023, Slovenia) [45]	Teeth (permanent/deciduous)	Full demineralisation extraction	Lower yield in deciduous/non-adult teeth	Prioritise adult permanent teeth	Permanent adult teeth have the highest yield	Small sample size
40	Seiberle et al. (2022, Switzerland) [46]	Blood/saliva/semen	Multiple swab types & volumes	Operator variability	Surface-specific wetting volumes	ForSaf & ForEvi swabs performed best	Short-term storage
41	Elwick et al. (2022, USA) [25]	Cartridge casings	Soaking vs rinse-and-swab	Firing effects, low yield	Optimised chelators	Comparable recovery; material & firing status critical	High inter-analyst variation
42	Egger et al. (2022, Switzerland) [47]	Post-coital vaginal swabs	ForensiX SafeDry swabs	Sperm persistence variability	SafeDry drying system	Interpretable male profiles up to 48h	48h maximum
43	Biggin et al. (2022, Canada) [48]	Bloody snow	Filter paper or swab collection	Cold dilution & degradation	Filter paper collection + drying	Complete STR profiles achieved	Short 1h exposure
44	Hedman et al. (2021, Sweden) [49]	Trace DNA on surfaces	Standardised swabbing (angle/rotation/wetting)	High person-to-person variation	Trained protocol	Significant yield improvement on ridged/porous surfaces	Saliva stains only
45	McKinnon & Higgins (2021, Australia) [50]	Burned porcine bone	Supernatant retention after demineralisation	Burn-induced fragmentation	Retain EDTA supernatant	Significantly increased DNA yield	Porcine proxy
46	Jansson et al. (2020, Sweden) [51]	Cartridge cases	Nylon-flocked swabs, Chelex	Firing loss & mixtures	Nylon swabs + simplified protocol	Marked improvement in detection rate	Increased mixture complexity
47	Gosch et al. (2020, Germany) [52]	Firearm touch DNA	Nylon double-swab	Realistic handling variability	Rapid sampling post-contact	Shooter DNA variable by duration & wiping	Simulated firearms
48	Sanches & Schreier (2020, USA) [53]	Estuarine eDNA	Glass fibre filters + magnetic beads	Turbidity & inhibitors	Inhibitor removal steps	Best balance of yield, time & cost	Tank-based spiking
49	Mishra et al. (2020, India) [54]	Touch DNA	Various collection & extraction methods	Low quantity & contamination	Sophisticated kits & careful handling	Valuable for source attribution when handled properly	Narrative review
50	Burrows et al. (2017, South Africa) [5]	Saliva samples	Novel FDL buffer vs Oragene	Long-term room-temp storage	Economical buffer + phenol-chloroform	Full STR profiles after 24 months	Small n = 10
51	Brandstätter et al. (2006, Austria) [55]	mtDNA control region	Chelex extraction, redundant sequencing	Data quality & errors	LIMS + double evaluation	High-quality population database	Relatively small n = 273
52	Choudhary et al. (2017, India) [56]	Animal bone DNA	Full demineralisation + organic extraction	Mineral barriers in aged bones	Complete dissolution protocol	High yield across fresh/old/buried bones	Animal samples
53	Stoop et al. (2017, Switzerland) [57]	Touch DNA on cotton	SceneSafe minitapes, phenol-chloroform	Extraction efficiency	Direct extraction from tape	Tape recovered 2–3× more DNA than swabs	Cotton fabric only
54	Hinlo et al. (2017, Australia) [58]	Water eDNA	CN filters + DNeasy, ethanol preservation	Filter clogging & inhibitors	Filtration + cold/ethanol storage	Highest yield and efficiency	Lab + limited field
55	Eychner et al. (2017, USA) [59]	Chewing gum	QIAamp & DNA IQ kits	Gum base complexity	Whole gum or wet swab	Good recovery with optimised kits	Environmental history unknown
56	Ghaheri et al. (2016, Iran) [60]	Whole blood	Optimised salting-out & phenol-chloroform	Toxicity & variable yield	Moradi salting-out method	Best balance of yield, purity & safety	Single donor
57	Mawlood et al. (2015, UK) [61]	Saliva stains	ForensiX active drying swabs	Storage degradation in trace amounts	Active drying tubes	2–4× higher long-term recovery	Glass slides only

**Table 3:** Initial processing protocols and challenges by biological evidence type.

Evidence Type	Initial Processing Protocols	Main Early Challenges	Yield-Preserving Strategies
Blood	Locate, swab or collect substrate, dry promptly, store cool, avoid excess surrounding debris [12,29,43,60,62].	Moisture, dilution, UV, transport degradation [12,29,60,62]	Rapid drying and cold storage preserved full STR profiles in snow and long persistence indoors [12,29,60].
Semen	Swab with assay-compatible buffer; preserve the same DNA stain where possible [33,62].	Faint stains, relocation difficulty, and sample depletion [33,62].	Transfer-paper mapping sped relocation ∼5.5-fold and still allowed full STR profiles [6,62].
Saliva	Moist swabbing with SERATEC buffer or similar non-destructive workflow [5,17,62].	Time-dependent nuclear degradation and denim-associated interference [12,17,62].	Saliva remained detectable to 180 days; mtDNA stayed recoverable despite partial nuclear loss [12,55].
Touch DNA	Single or double swabbing, tape-lifting, standardised pressure and angle, active drying [6,9,16,57].	Low quantity, dropout, mixtures, shedder variability, transfer [12].	SDS outperformed water on glass; tape often outperformed swabs; optimised swabbing increased yield [6,12].

### 2.3 Study selection

**Fig. 1:**
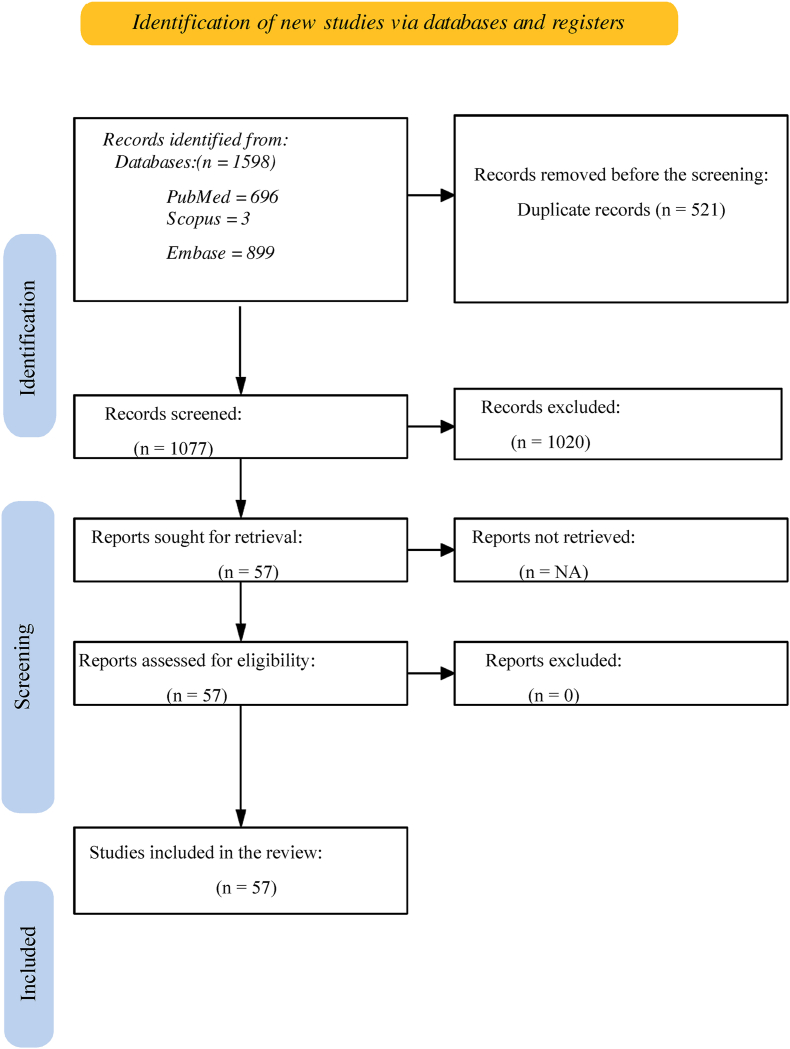
PRISMA-ScR selection process for diagram.

Full-text English articles that addressed one or more forensic science methods used directly in protocols and the challenges in managing multi-source biological evidence (blood, semen, saliva, touch DNA) at the crime scene were considered. Studies were screened, and duplicates were removed; some studies were resolved through two independent reviews when there was some conflict between studies. Fig. 1 is the flow of the PRISMA-ScR. In Fig. 1, the PRISMA-ScR study selection process is illustrated (see Table 3).

### 2.4 Collating, summarising, and reporting the results

To chart the data, parameters such as author(s)/year/region, study design, evidence type(s), collection and preservation protocols, key challenges, strategies to maximise yield and chain of custody, main findings, limitations, and future scope/recommendations were extracted and tabulated. A thematic narrative synthesis was used, along with descriptive statistics summarising the studies' characteristics, geographic distribution, and types of evidence. The review is based on 57 distinct sources, predominantly from high-income countries.

• Swabbing and tape-lifting techniques for touch and trace DNA recovery [6,9,16].
• Preservation and storage protocols using drying, freezing, and chemical buffers [4,12,20].
• Visualisation and documentation aids for targeting biological stains [7,19,33].
• Anti-contamination and automated extraction strategies to support chain-of-custody integrity [13,24,36].
• Evidence-specific optimisations for blood, semen, saliva, and degraded samples [17,18,25].

## 3 Results

The scoping review synthesised findings from 57 studies examining protocols for initial crime scene management and the preservation of multi-source biological evidence (blood, semen, saliva, and touch DNA). Most studies were experimental or comparative; mock exhibits and controlled depositions were studied most often, followed, less frequently, by real casework or aquatic recoveries.

Discussions of DNA yield and profile quality improvements resulting from forensic collection and preservation protocols are objective and quantifiable. Optimised double-swab techniques (wet + dry; sterile water, PBS, 2% SDS) and adhesive tape lifting and substrate-specific approaches have consistently achieved higher cell recovery and lower contamination rates than conventional visual inspection and basic swabbing [6,9,16]. Preservation strategies include immediate air-drying, active drying tubes, freezing (−20°C to −80°C), Longmire's buffer, and ethanol or solid NaCl, all of which reduce degradation and facilitate downstream STR analysis or mitochondrial genome (mtDNA) sequencing [4,12,32].

Cells on tape lifts are counted using the visualisation tool of Diamond Nucleic Acid Dye. For burned bone, quantitative colour analysis (Lab) increases the targeting and predictive value for genotyping success [7,19]. Integrity of the chain of custody is vital, and anti-contamination frameworks that include PPE, changing gloves, UV/bleach decontamination, negative controls, elimination databases, and separate tamper-evident packaging are essential [24,36,41]. For samples with small amounts of template, automated extraction platforms (such as Maxwell RSC and AutoMate Express) and carrier additives (such as salmon sperm DNA) further maximise OMICS (multi-omics, i.e. genomic, transcriptomic, and related molecular profiling) data yields [20,22].

### 3.1 Scientific reliability

Under controlled conditions, the techniques reviewed demonstrate high analytical reliability but exhibit environmental-, substrate-, and operation-dependent variability. Double-swab and tape-lifting procedures both yield reproducible gains in DNA recovery, but there is considerable variation among operators [46,49]. The yield reductions and degradation indices are statistically significant during storage in unregulated museum or aquatic settings over months to years, with many sample types demonstrating good stability with preservation protocols (cold storage or chemical buffers) (p < 0.001), [4,12]. Anti-contamination methods aid forensic admissibility, with validation, visualisation, and direct PCR approaches improving objectivity [9,24]. Although automated and AI-enabled cell counting offer advantages in speed, implementation is hindered by standardisation issues [7].

### 3.2 Investigative contributions

These protocols significantly improve trace evidence recovery, enable reliable mixture deconvolution, aid activity-level interpretation, and enhance chain-of-custody documentation. The use of multi-method convergence (e.g., swabbing + visualisation + optimised preservation) contributes to the completion of STR profiles and minimisation of allele drop-out, and therefore to more probative forensic intelligence [7,17,18]. Quick processing and separate packaging reduce secondary transfer, and strategies that focus on mtDNA yield good results with highly degraded samples [4,23].

### 3.3 Limitations and future directions

Most studies were conducted on lab-made mock scenes with limited replicates, single donors, or limited storage time, and were not generalisable to dynamic real crime scenes. Representation in low and middle-income countries is growing but still low. The biggest problems remain: porous substrate absorption, aquatic degradation, burned bone complexity, and stochastic effects in the presence of a low-copy template.

Large-scale multi-site field validations, automated visualisation and extraction pipelines, low-cost tools for resource-limited settings, and standardised international guidelines for aquatic and wet evidence are areas of research that should be prioritised in the future, as well as integration with NGS/miniSTR panels for highly degraded samples [4,7,18].

## 4 Discussion

This scoping review aims to summarise information from 57 studies (2015–2025) regarding protocols and challenges for the processing and preservation of multi-source biological evidence (blood, semen, saliva and touch DNA) at the initial crime scene, to maximise the downstream yield of forensic deoxyribonucleic acid (DNA) (quantification, short tandem repeat [STR] profiling, mitochondrial DNA [mtDNA] sequencing) and chain-of-custody integrity.

One of the key issues is the competing requirements of trace-evidence sensitivity and operational requirements. Low-template stochastic effects, allelic dropout and secondary transfer are common problems with trace (touch) DNA. The presence of a shedder has a significant impact on yield (p < 0.001), while the porosity/environmental exposure of the DNA substrate further modulates the recovery [16,18]. Both saliva and semen have been shown to undergo time-dependent degradation, with significant degradation after 90–180 days at room temperature [4,17], and to undergo extensive loss of nuclear DNA in aquatic environments after 21 days [4]. Cases involving blood on snow or burned bone cause issues with dilution and fragmentation of the target DNA template, and inhibition of the PCR reaction [19,48], making immediate action essential.

Double-swab (wet and dry) methods with optimised solutions (phosphate-buffered saline [PBS], Triton X-100, or 2% sodium dodecyl sulfate [SDS]) and controlled wetting volume (∼45 μL), swabbing pressure and rotation, to improve recovery across surfaces [6,49]. Adhesive tape lifting with visualisation dyes (e.g., Diamond Nucleic Acid Dye) is superior to swabbing for touch DNA on non-porous and fabric surfaces in terms of the number of complete DNA profiles obtained and contamination levels [7,9]. Acid phosphatase (AP) press testing using Saral Transfer Paper is used for semen localisation [33]. For cartridge casings, a chelator-assisted rinse-and-swab technique is used to recover DNA [25].

Carriers [20]. Separate tamper-evident paper-bag packaging reduces secondary transfer, thereby lowering by up to 10% the risk of erroneous attribution to a foreign, co-packaged source [8,35,41].

Anti-contamination and chain-of-custody protocols include complete personal protective equipment (PPE) use with glove changes, ultraviolet (UV)/bleach decontamination, negative controls, an elimination database, and detailed documentation, which are essential to prevent the introduction of extraneous DNA, to ensure DNA integrity, and to provide the means for follow-up review and correction where issues arise [13,14,36].

The techniques described demonstrate high scientific reliability under controlled conditions, but this reliability is reduced by inter-operator variation, substrate interactions, and the dynamic nature of the crime scene [46,51]. Preservation is suitable for short-to medium-term storage, but unregulated storage results in significant degradation [12]. Direct PCR and automated extraction increase workflow speed but are dependent on the workflow [9,22].

## 5 Conclusion

This scoping review of 57 published peer-reviewed studies (from 2015 to 2025) outlines current protocols and ongoing issues related to the initial crime scene management and preservation of multi-source biological evidence, such as blood, semen, saliva, and trace (touch) DNA, with a clear focus on maximising downstream deoxyribonucleic acid (DNA) yield as well as maintaining chain-of-custody integrity for evidentiary admissibility.

There are a set of core practices synthesised from the evidence; (1) double-swab (wet + dry) collection, or adhesive tape lifting using buffered solutions (e.g., phosphate buffered saline [PBS] or 2% sodium dodecyl sulfate [ SDS]) with the addition of visualisation agents such as Diamond Nucleic Acid Dye; (2) immediate stabilisation using air-drying or active-drying systems; (3) chemical preservation (Longmire's buffer, ethanol, solid NaCl) or cold storage (−20°C to −80°C) with the addition of carrier nucleic acids (e.g., salmon sperm DNA); and (4) rigorous anti-contamination measures, including personal protective equipment (PPE), elimination databases, negative controls, and independent tamper-evident packaging. All of these techniques have been shown to increase DNA recovery, reduce allelic dropout and stochastic artefacts, improve the quality of STR and mtDNA profiles, and decrease secondary transfer. Beyond these gains, this package of measures also provides the means for follow-up review and corrective action when problems arise, supporting continuous improvement and maximising the evidentiary value of biological evidence both in the present and as practice evolves.

Even with these advances, there are still significant challenges, such as low-template stochasticity, shedder inter-individual variability, matrix effects from substrates, rapid environmental degradation (particularly in aquatic, humid, and/or thermal environments), operator variability, and indirect contamination risks. Most studies are laboratory- or mock-scene-based, limiting ecological validity and generalisability, especially in low- and middle-income countries (LMIC) contexts.

The literature confirms that a rapid, minimal-handling, substrate-specific collection method, combined with swift, condition-specific preservation and rigorous chain-of-custody procedures, is the current best practice. These strategies need to be prioritised and spread across multiple field sites, automated, harmonised across countries, and implemented in a context-sensitive way to achieve equitable, high-quality results in operational settings.

## Funding

Not applicable.

## CRediT authorship contribution statement

**Mayur Sudhir Balbudhe:** Conceptualization, Data curation, Investigation, Methodology, Validation, Writing – original draft, Writing – review & editing. **B Suresh Kumar Shetty:** Project administration, Supervision, Validation, Visualization, Writing – review & editing. **Saroj Babar:** Project administration, Supervision, Validation, Writing – review & editing. **Akshita Sinha:** Data curation, Formal analysis. **Sahil Gupta:** Data curation, Formal analysis.

## Declaration of competing interest

The authors declare that they have no known competing financial interests or personal relationships that could have appeared to influence the work reported in this paper.

## References

[1] Pfefferli P. (2015).

[2] Fox R.H., Cunningham C.L. (1973).

[3] Baxter E. (2015).

[4] Shahzad M., De Maeyer H., Salih G.A., Nilsson M., Haratourian A., Shafique M., Shahid A.A., Allen M. (2024 Feb 23). Evaluation of storage conditions and the effect on DNA from forensic evidence objects retrieved from lake water. Genes.

[5] Burrows A.M., Ristow P.G., D'Amato M.E. (2017 Dec 1). Preservation of DNA from saliva samples in suboptimal conditions. Forensic Sci. Int.: Genet. Suppl. Ser..

[6] Schulte J., Rittiner N., Seiberle I., Kron S., Schulz I. (2023 Jul). Collecting touch DNA from glass surfaces using different sampling solutions and volumes: immediate and storage effects on genetic STR analysis. J. Forensic Sci..

[7] Madden I., Taylor D., Mitchell N., Goray M., Henry J. (2024 May 1). Predicting probative levels of touch DNA on tapelifts using diamond™ nucleic acid dye. Forensic Sci. Int. Genet..

[8] Stella C.J., Meakin G.E., van Oorschot R.A.H. (2022). DNA transfer in packaging: attention required. Forensic Sci Int Genet Suppl Ser..

[9] Asiri Z., Rameshbabu S., Kumar S., Alsaleh A., Ouanes K., Messaoudi S.A. (2025 Oct). Adhesive tape versus swabbing for touch DNA collection: a direct PCR approach with the VeriFiler express kit. J. Forensic Legal Med..

[10] Arksey H. (2005). Scoping studies: methodological framework. Int. J. Soc. Res. Methodol..

[11] Aromataris E. (2024). JBI Manual for Evidence Synthesis.

[12] Jeromelj T., Leskovar T., Zupanič Pajnič I. (2025 Jan 11). The impact of storage conditions on DNA preservation in human skeletal remains: a comparison of freshly excavated samples and those stored for 12 years in a museum depot. Genes.

[13] Hymus C.M., Cooper P.L., Rye M.S. (2025 Jan 1). Demonstration of potential DNA contamination introduced by laboratory consumables using fluorescein. Forensic Sci. Int. Genet..

[14] Tiplamaz S., Ömeroğlu Ulu Z., Colak B., Asliyüksek H., Şahin F. (2025). Forensic identification using airDNA: a preliminary study on the collection, isolation, amplification and sequencing of human DNA from air samples. Turk. J. Med. Sci..

[15] Allan E.A., Shaffer M.R., Kelly R.P., Parsons K. (2025 Oct 30). Optimising target-to-total DNA ratio in eDNA studies: effects of sampling, preservation, and extraction methods on single-species detection. PeerJ.

[16] Saamia V., Yudianto A., Nurjayadi M. (2024 Dec 3). Touch DNA viability on various substrates from different shedder levels. Med. J. Indonesia.

[17] Zapico S C., Roca G. (2024 Jun). A spit in time: identification of saliva stains and assessment of total DNA recovery up to 180 days after deposition. Forensic Sci. Med. Pathol..

[18] Arsenault H., Kuffel A., Dugard P., Nic Daeid N., Grey A. (2025 Jan 1). Trace DNA and its persistence on various surfaces: a long-term study investigating the influence of surface type and environmental conditions–part two, non-metals. Forensic Sci. Int. Genet..

[19] Macias E., Hartline K., Buzzini P., Hughes S. (2024 May). Quantitative colour analysis of burned bone to predict DNA quantity, quality, and genotyping success. J. Forensic Sci..

[20] Oechsle C.M., Paul T.A., Seichko J.D., Worst T.J. (2024 Mar 1). Salmon sperm DNA increases sample recovery from cotton swabs. Forensic Sci. Int. Genet..

[21] Meakin G.E., Hare K., Robinson N., Poulsen F., Hitchcock C., Best M., Griffin B., Raymond J. (2024 Apr 30). Initial testing of an applicator prototype to facilitate forensic DNA recovery by tapelifting. Aust. J. Forensic Sci..

[22] Griffin A., Kirkbride K.P., Henry J., Painter B., Linacre A. (2023 Nov 1). Comparison of three DNA extraction methods tested on illicit drug-related powders. Forensic Sci. Int. Genet..

[23] Haarkötter C., Vinueza‐Espinosa D.C., Gálvez X., Saiz M., Medina‐Lozano M.I., Lorente J.A., Álvarez J.C. (2023 Oct). A comparison between the petrous bone and tooth, the femur and tibia, and DNA analysis from degraded skeletal remains. Electrophoresis.

[24] Comment D., Gouy A., Zingg C., Zieger M. (2023 Jul 1). A holistic approach for the selection of forensic DNA swabs. Forensic Sci. Int..

[25] Elwick K., Gauthier Q., Rink S., Cropper E., Kavlick M.F. (2022 Jul 1). Recovery of DNA from fired and unfired cartridge casings: comparison of two DNA collection methods. Forensic Sci. Int. Genet..

[26] Harnpicharnchai P., Pumkaeo P., Siriarchawatana P., Likhitrattanapisal S., Mayteeworakoon S., Ingsrisawang L., Boonsin W., Eurwilaichitr L., Ingsriswang S. (2023 Jun 29). AirDNA sampler: an efficient and simple device enabling high-yield, high-quality airborne environment DNA for metagenomic applications. PLoS One.

[27] Yuliarti K., Mansyur M., Timan I.S., Ariani Y., Sidhiarta I.G., Ramadhani N.T., Prakoso N.M., Sjarif D.R. (2023 Jul 11). DNA quality from buccal swabs in neonates: comparison of different storage times—Medical Journal of Indonesia.

[28] de Arellano Sánchez J.A., Ortiz J.M., Quintero A.L., Pfeiffer H., Vennemann M., Bauer H. (2023 Mar). Comparing preservation substrates under field conditions for efficient DNA recovery in bone. Int. J. Leg. Med..

[29] Holzschuh A., Koepfli C. (2022 Mar 15). Tenfold difference in DNA recovery rate: systematic comparison of whole blood vs. dried blood spot sample collection for malaria molecular surveillance. Malar. J..

[30] Zareef I., Rathore A.W., Zaheen U., Riaz A., Rakha A., Munawar A. (2025 Sep). A model research study on persistence, recovery and analysis of trace DNA under fingernails of drowned bodies. Forensic Sci. Int..

[31] Van Thanh Le T., Vuong T.B., Le G.H., Do D.M. (2025 Nov 3). Scraping enhances microbial DNA recovery over swabbing in sensitive facial skin: a pilot study of 10 patients. Sci. Rep..

[32] Tomke S.A., Buland E.N., Price S.J. (2025). Evaluation of preservation and extraction methods on eDNA yield and detection probability after long-term storage. Mol. Ecol. Resour..

[33] Thong Z., Wee A.P., Heng B., Syn C.K. (2025 Sep 12). Rapid documentation of possible semen stains for forensic DNA profiling. Genes.

[34] Lisman D., Ossowski A., Tołoczko-Grabarek A., Kozłowski M., Cymbaluk-Płoska A. (2025 Sep 12). Forensic DNA recovery from FFPE tissue using the maxwell® RSC Xcelerate Kit: optimisation, challenges, and limitations. Genes.

[35] Lee Y.S., Syn C.K. (2025 Jul 28). DNA transfer between items within an evidence package. Genes.

[36] Gaskell M., Guiness J., Sullivan K. (2025 Aug 1). Understanding and mitigating the risks that environmental DNA contamination poses to the recovery of forensic evidence from victims and suspects of rape and sexual assault. J. Forensic Legal Med..

[37] Santamaria P., Ghuman M., Nibali L. (2025 Aug 1). Impact of optimised sample preparation method on DNA recovery from subgingival plaque in a population with periodontitis. Arch. Oral Biol..

[38] Kravanja P., Golob A., Concato M., Leskovar T., Pajnič I.Z. (2025 Jun 1). Effects of different environmental factors on preservation of DNA in petrous bones: a comparative study of two Slovenian archaeological sites. Forensic Sci. Int..

[39] Priya V., Sudhakaran R. (2025 May 5). A novel approach for DNA extraction of white spot syndrome virus detection in penaeid shrimp. Sci. Rep..

[40] Pajnič I.Z., Leskovar T. (2025 Apr 1). How to store the bone powder left after extraction for future analysis. Forensic Sci. Int..

[41] Castella V., Hicks T., Samie L., Basset P. (2025 Mar 1). A case of contamination by indirect DNA transfer in a sexual assault case: a taste of déjà vu?. Forensic Sci. Int..

[42] Gontijo J.B., Valverde Firmino G., Mandro J.A., Miranda Reis A.L., Bieluczyk W., Feitosa Fernandes J.C., Barbosa de Camargo P., Mazza Rodrigues J.L., Mui Tsai S., Vidal-Torrado P. (2025 Jan 6). An effective DNA extraction protocol optimised for tropical swamp peat samples. Microbiol. Spectr..

[43] Samsonova J.V., Saushkin N.Y., Voronkova V.N., Stolpovsky Y.A., Piskunov A.K. (2025 Aug). Optimisation of total DNA extraction from dried blood samples. Biochem. Genet..

[44] Brown M.E., Ottati S., Trivellone V. (2025 May). A non-destructive, fast, inexpensive, non-toxic chelating resin-based DNA extraction protocol for insect voucher specimens and associated microbiomes. J. Insect Sci..

[45] Leskovar T., Pajnič I.Z. (2023 Dec 1). Comparative analysis of DNA preservation in permanent and deciduous teeth of adults and non-adults: implications for archaeological and forensic research. Forensic Sci. Int..

[46] Seiberle I., Währer J., Kron S., Flury K., Girardin M., Schocker A., Schulz I. (2022). Collaborative swab performance comparison and the impact of sampling solution volumes on DNA recovery. Forensic Sci. Int. Genet..

[47] Egger S., Vöhringer C., Währer J., Schulz I. (2022 Jul 1). Comparison of forensic swabs for intravaginal sampling. Sci. Justice.

[48] Biggin M.R., Albrecht I., Novroski N.M. (2022 Mar 1). Assessing DNA recovery and profile determination from bloody snow. Sci. Justice.

[49] Hedman J., Akel Y., Jansson L., Hedell R., Wallmark N., Forsberg C., Ansell R. (2021 Jul 1). Enhanced forensic DNA recovery with appropriate swabs and optimised swabbing technique. Forensic Sci. Int. Genet..

[50] McKinnon M., Higgins D. (2021 Mar 1). Comparison of bone demineralisation procedures for DNA recovery from burned remains. Forensic Sci. Int. Genet..

[51] Jansson L., Forsberg C., Akel Y., Dufva C., Ansell C., Ansell R., Hedman J. (2020 Sep 1). Factors affecting DNA recovery from cartridge cases. Forensic Sci. Int. Genet..

[52] Gosch A., Euteneuer J., Preuß-Wössner J., Courts C. (2020 Sep 1). DNA transfer to firearms in alternative realistic handling scenarios. Forensic Sci. Int. Genet..

[53] Sanches T.M., Schreier A.D. (2020 May 21). Optimising an eDNA protocol for estuarine environments: balancing sensitivity, cost and time. PLoS One.

[54] Mishra I.K., Singh B., Mishra A., Mohapatra B.K., Kaushik R., Behera C. (2020 Apr 1). Touch DNA as forensic aid: a review. Indian J. Forensic Med. Toxicol..

[55] Brandstätter A., Niederstätter H., Pavlic M., Grubwieser P., Parson W. (2007 Mar 2). Generating population data for the EMPOP database—an overview of the mtDNA sequencing and data evaluation processes considering 273 Austrian control region sequences as example. Forensic Sci. Int..

[56] Choudhary S, Rathore P, Boyal PK, Meena D, Yadav R, Mehta SC, Kataria AK. Bone DNA Extraction was Collected from Different sources/conditions.

[57] Stoop B., Defaux P.M., Utz S., Zieger M. (2017 Nov 1). Touch DNA sampling with SceneSafe fast™ minitapes. Leg. Med..

[58] Hinlo R., Gleeson D., Lintermans M., Furlan E. (2017 Jun 12). Methods to maximise recovery of environmental DNA from water samples. PLoS One.

[59] Eychner A.M., Schott K.M., Elkins K.M. (2017 Jan). Assessing DNA recovery from chewing gum. Med. Sci. Law.

[60] Ghaheri M., Kahrizi D., Yari K., Babaie A., Suthar R.S., Kazemi E. (2016 Mar 31). A comparative evaluation of four DNA extraction protocols from a whole blood sample. Cell. Mol. Biol..

[61] Mawlood S.K., Alrowaithi M., Watson N. (2015 May). Advantage of ForensiX swabs in retrieving and preserving biological fluids. J. Forensic Sci..

[62] Medina-Paz F., Kuba B., Kryvorutsky E., Roca G., Zapico S.C. (2024). Assessment of blood and semen detection and DNA collection from swabs up to three months after deposition on five different cloth materials. Int. J. Mol. Sci..

